# Hydrogen Damage Behavior of X80 Pipeline Steel Under AC Interference

**DOI:** 10.3390/ma18245487

**Published:** 2025-12-05

**Authors:** Tong Li, Zhihui Li, Kejun Jiang, Yuxiang Cai, Wan Sun, Ziyong He, Jun Zhao, Tao Cao, Junjun Jin, Wenjing Chen, Guoqing Gou

**Affiliations:** 1 School of Materials Science and Engineering, Xihua University, Chengdu 610039, China; lit072511@163.com (T.L.); li17635033834@163.com (Z.L.); wusheng050177@163.com (T.C.); 2 Sichuan Provincial Engineering Research Center of Intelligent Welding for Energy Equipment, Chengdu 610039, China; 3 Key Laboratory of Advanced Technologies of Materials, Ministry of Education, School of Materials Science and Engineering, Southwest Jiaotong University, Chengdu 610031, China; kkj720@my.swjtu.edu.cn (K.J.); 13440162476@163.com (Y.C.); jinjun2358@163.com (J.J.); 4 Sanan-ST Microelectronics Co., Ltd., Chongqing 401332, China; wan.sun@sast-semi.com; 5 Dongfang Electric Machinery Co., Ltd., Deyang 618000, China; hezy6887@dongfang.com (Z.H.); runner_wxlz@163.com (J.Z.)

**Keywords:** X80 pipeline steel, AC hydrogen, SSRT, welding joint

## Abstract

X80 pipeline steel is a key material in the field of oil and gas transportation. Its damage behavior in a hydrogen-filled environment directly affects pipeline safety. In this study, through hydrogen permeation experiments and slow strain rate tensile tests, the electrochemical responses and hydrogen-induced cracking behaviors of X80 base metal and welded joints under hydrogen filling conditions in both AC and DC were systematically compared. The results show that when the base material is filled with hydrogen at 20 mA/cm^2^ AC, the hydrogen permeation flux is the largest, and the overall hydrogen permeation parameter of the welded joint is lower than that of the base material. High-frequency polarization promotes hydrogen permeation, but anodic corrosion products at high current densities can impede hydrogen entry. The slow strain rate tensile test further confirmed that the mechanical properties of the material declined more significantly under direct current hydrogen charging, and the sensitivity to stress corrosion cracking was higher. Under alternating hydrogen charging conditions, due to the alternating effects of hydrogen charging at the cathode and corrosion at the anode, a relatively low hydrogen embrittlement sensitivity is exhibited.

## 1. Introduction

With the vigorous development of the world economy, the demand for energy is growing. Among various energy transportation modes, pipeline transportation has become the most popular means of oil and gas transportation due to its advantages of low cost, large transportation volume, low energy consumption and high safety. With the continuous updating and iteration of pipeline steel, the current research on pipeline steel gradually focuses on improving more excellent mechanical properties such as stiffness and strength. However, with the improvement of pipeline steel grade and the improvement of material mechanical properties, its corrosion resistance has shown a downward trend [1]. This trend is particularly evident, considering that most oil pipelines are buried deep in the underground environment. With the extension of the service time of the pipeline, it is more susceptible to corrosion [2,3,4], which leads to corrosion pits, even cracks, and ultimately leads to pipeline failure. Therefore, the research on pipeline materials not only needs to pay attention to the improvement of its mechanical properties, but also needs to pay attention to the corrosion problems it faces in practical applications. In 1985, X80 pipeline steel was successfully developed by Mannesmann Company in Germany [5]. In the same year, it was included in the API standard, which has a history of 39 years. Subsequently, the establishment of the Canadian NOVA Gas Transmission system played an important role in promoting the application of X80 pipeline steel in high-strength pipeline steel. [6,7]. So far, the total length of X80 pipeline steel pipeline that has been built around the world is about 8000 km. With the application and promotion of this technology, X80 pipeline steel plays an increasingly important role in the field of pipeline construction [8]. At present, about 17,000 km of X80 oil and gas pipeline have been built in China, which makes the material research and development, application production and pipeline construction of X80 pipeline steel reach the leading level in the world. As the most widely used material, the safety of X80 pipeline steel has attracted much attention. With the increasing popularity of ‘public corridors’ formed by crossing and paralleling pipelines with high-voltage wires and electrified railways [9], the risk of AC corrosion and hydrogen damage caused by the AC escaping into the soil has increased significantly, which greatly increases the possibility of stress corrosion cracking of pipelines [10,11,12]. Once the pipeline breaks down, it will seriously threaten the safety, economy and environment of personnel. It is of great significance to carry out relevant corrosion and hydrogen damage mechanism research to ensure the long-term safe operation of pipelines.

In early studies, scholars found that at the same current density, the corrosion caused by AC stray current interference accounted for only 1% compared with the same amount of DC interference. Therefore, for buried metal structures, the corrosion hazards caused by AC stray current are generally considered to be negligible [13,14]. However, with the rapid development of power networks and electrified railways, several corrosion accidents of buried pipelines caused by AC stray current have occurred in Germany, the United Kingdom and North America [15]. In recent years, professionals in the field of corrosion protection of oil and gas pipelines in China have also observed the phenomenon of AC stray current corrosion of buried pipelines for many times. Domestic and foreign scholars have extensively studied the corrosion phenomenon of X80 pipeline steel caused by AC. The research results of Dae-Kyeong Kim [16] show that when the AC with low current density (2 mA/cm^2^) is applied, the corrosion rate of carbon steel is smaller when the cathodic protection potential of −1100 mV SCE is used. However, as the current density gradually increased to 10 mA/cm^2^, the corrosion rate of carbon steel increased significantly. The results of Fu [17] showed that uniform corrosion of carbon steel occurred at low current density. However, when the current density reached 50 mA/cm^2^, the passivation film on the surface completely disappeared and obvious local corrosion occurred. Chin [18] found that the application of AC will change the shape of polarization curve of low carbon steel in alkaline environment. With the increase in current density, the self-corrosion current density increases, and the self-corrosion potential shifts negatively, which reduces the corrosion resistance of the material. Min Zhu et al. [19] showed that the increase in AC density would accelerate the corrosion rate. When the current density reached 3 mA/cm^2^, X80 pipeline steel began to display the obvious stress corrosion cracking phenomenon. On the other hand, high-strength steel is more likely to experience hydrogen-induced failure than low-strength steel [20,21,22]. Peng XY et al. [23] studied the hydrogen damage behavior of pipeline steels with different steel grades. It was found that both high-grade pipeline steel and low-grade pipeline steel showed enhanced corrosion activity after hydrogen charging. Under the same hydrogen charging conditions, high-grade pipeline steel has higher density traps than low-grade pipeline steel, which may make high-strength steel more susceptible to hydrogen-induced cracking. Chuan He et al. [24] found that hydrogen can accelerate the anodic dissolution of steel, thereby enhancing the corrosion sensitivity, and with the increase in hydrogen charging time and current density, the corrosion sensitivity of steel increases gradually. Jia Yu Zhou et al. [25] studied the effect of AC interference on the hydrogen evolution reaction of X80 pipeline steel. The results show that the hydrogen evolution reaction may occur on X80 pipeline steel under AC conditions. When the current density exceeds the critical value of approximately 10–20 mA/cm^2^, the dominant cathodic reaction on the X80 steel shifts from the oxygen reduction reaction to the hydrogen evolution reaction. Lalvani et al. [11,12] interfered with carbon steel immersed in 3.3% NaCl solution with positive half-wave, negative half-wave and full-wave sinusoidal AC. The results showed that the AC corrosion rate of the three waveforms increased with the increase in peak voltage, among which the full-wave corrosion rate was the largest and the negative half-wave corrosion rate was the smallest. The failure mechanism of hydrogen embrittlement is largely influenced by many factors, including chemical composition, microstructure, density and type of hydrogen traps, strength level of steel, stress and environmental conditions [26,27,28,29,30,31,32].

However, up to now, research on the hydrogen damage behavior of this steel grade under alternating current interference has been relatively limited. To fill this research gap, this paper focuses on the hydrogen damage behavior of X80 pipeline steel under alternating current interference and systematically explores the stress corrosion cracking behavior of the base material and welded joints of X80 pipeline steel. Through in-depth research on the anodic corrosion and cathodic hydrogen charging behavior of X80 pipeline steel pipes under alternating current conditions, this paper aims to provide data support for the corrosion maintenance and prevention of pipelines. This research holds significant engineering significance for the operational safety of pipelines.

## 2. Materials and Methods

### 2.1. Sample Preparation and Characterization

**Figure 1:**
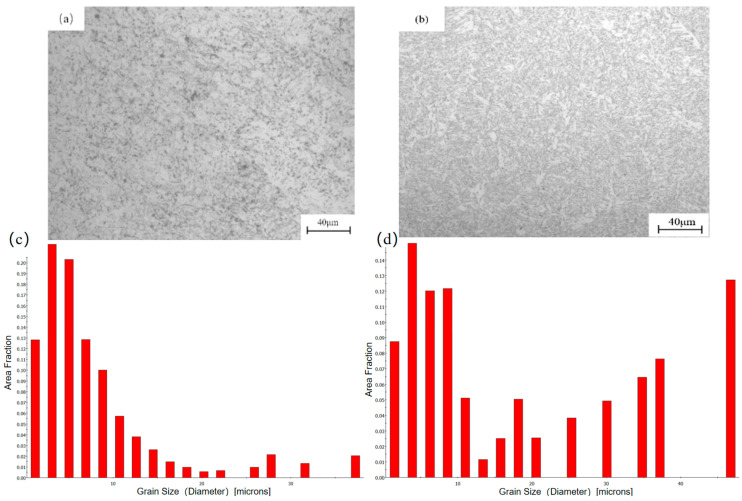
Microstructure of X80 pipeline steel base metal and welded joint: (**a**,**b**) metallographs; (**c**,**d**) grain size distributions.

In the experiment, X80 pipeline steel supplied by Southwest Pipeline Company (Chengdu, China) was selected as the research object, and the girth welding technology of pipeline was used in the welding experiment. Its size is D1016 mm × 15.3 mm. The pipeline adopts a V-groove of 47° ± 3°, retains a blunt edge of 1.0 ± 0.5 mm, and the gap between the two pipelines is 3.5 ± 1.0 mm. We selected the GTAW + FCAW-S hybrid welding method commonly used in practical engineering applications. Table 1 shows the main chemical composition of X80 pipeline steel base metal. Figure 1a shows the microstructure of X80 pipeline steel base metal. The microstructure of the base metal shows a fine and uniform grain structure, which is mainly composed of AF and GB, and there are M/A components at the grain boundary. This microstructure shows that the X80 pipeline steel base metal has good microstructure characteristics. During the welding process, the microstructure and composition of the welded joint are significantly affected by the heat input. As shown in Figure 1b, the welded joint of X80 pipeline steel is mainly composed of ferrite, which is mainly acicular ferrite and contains a small amount of polygonal ferrite. The high-density dislocation and precipitation in the welded joint will increase the strength and toughness of the material, but a small amount of polygonal ferrite may reduce the overall toughness level of the welded joint.

### 2.2. Hydrogen Permeation Experiment

**Figure 2:**
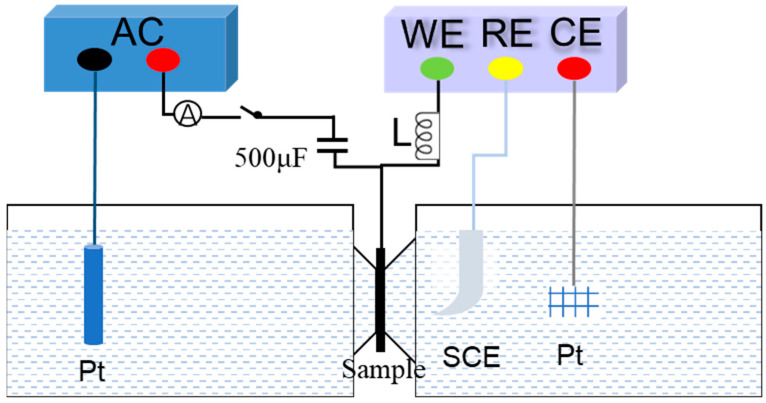
The schematic diagram of hydrogen permeation experiment method in double electrolytic cell.

A double electrolytic cell experimental device was used for the hydrogen permeation experiment. The device consists of an electrochemical electrolytic cell and a hydrogen charging cell. The specific structure is shown in Figure 2. The sample is a circular steel plate with a diameter of 30 mm and a thickness of 1 mm. The area exposed to the hydrogen-filled solution is 0.785 cm^2^. In order to avoid the influence of surface oxidation and corrosion on the experimental results after polarization in the oxidation tank, a nickel layer about 1 μm thick was pre-plated on the surface of the sample before the hydrogen permeation experiment. The electrochemical method was used for hydrogen charging. The left side of the sample was equipped with a hydrogen charging cell. The hydrogen charging solution was a mixed solution of 0.5 mol/L H_2_SO_4_ and 0.2 g/L CH_4_N_2_S. The right side of the sample was equipped with an electrolytic cell. The electrolyte was 0.1 mol/L NaOH solution. A constant potential of 300 mV was applied and constant potential polarization was used. The hydrogen evolution reaction occurs after the sample is energized as a cathode, and a part of the generated hydrogen atoms penetrates into the sample and is captured or diffused by internal defects. Hydrogen charging was initiated when the hydrogen permeation current density decreased to less than 1 μA/cm^2^. As the hydrogen charging progresses, the hydrogen permeation and diffusion eventually reach equilibrium, and the hydrogen permeation current density also reaches a steady state, thereby obtaining the hydrogen permeation curve. By recording the steady-state current density and the time required to reach the steady state, the hydrogen permeation parameters of the material can be calculated.

In addition to a systematic investigation of the hydrogen permeation behavior of X80 pipeline steel base metal under AC charging at 5~50 mA/cm^2^, supplementary experiments were conducted under DC and diode-rectified positive AC half-wave charging. For the welded joint samples, the main focus was on the hydrogen permeation properties of the welded joint samples under 5 and 20 mA/cm^2^ AC hydrogen charging. This helps to evaluate the hydrogen permeation performance of the three welding methods in a strongly acidic environment and an AC hydrogen charging environment. All alternating current (AC) signals employed in this study had a fixed frequency of 50 Hz.

### 2.3. Slow Strain Rate Tensile Test

**Figure 3:**
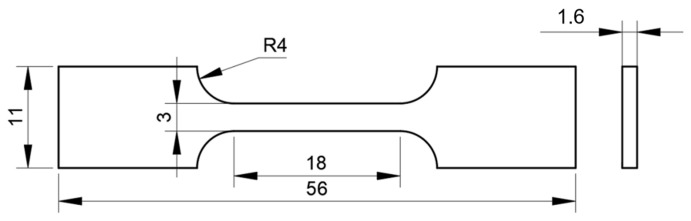
Slow strain rate tensile specimen size (mm).

**Figure 4:**
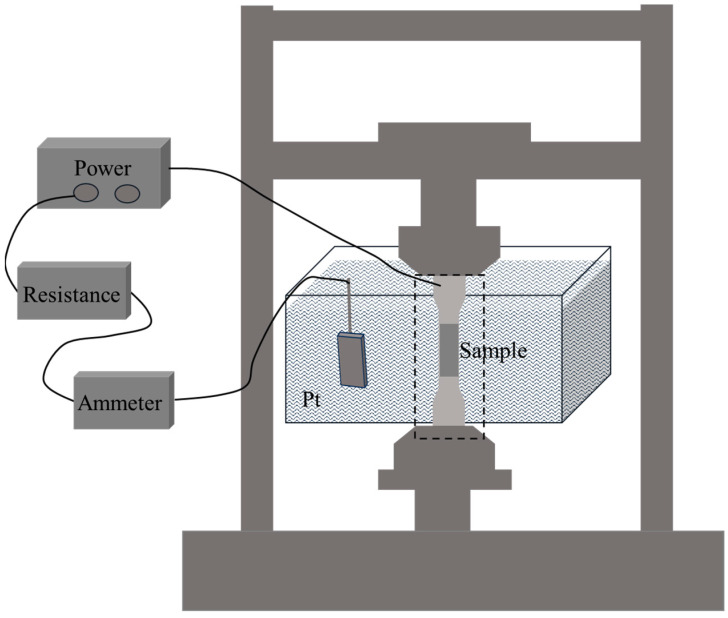
Slow strain rate tensile experimental device schematic diagram.

In this experiment, in situ electrolytic hydrogen charging method was used, and slow strain rate tensile test was carried out at the same time. The slow strain rate tensile specimens are flat in shape (see Figure 3 for sample size, standard: ISO 7539-7 [33]). The slow strain rate tensile tests were performed using a universal testing machine (model: HDW-50K, manufacturer: Chengdu Times Hongyu Testing Instrument Co., Ltd., Chengdu, China). The experiments were carried out in air and hydrogen-filled solution (0.5 mol/L H_2_SO_4_ + 0.2 g/L CH_4_N_2_S), respectively. The stretching rate was 10^−5^ s^−1^ and the experimental temperature was room temperature. Three parallel experiments were carried out under each experimental condition. The schematic diagram of the experimental device is shown in Figure 4. At the end of the experiment, the fracture morphology of the samples under different hydrogen charging conditions was observed by field emission scanning electron microscope to analyze the corrosion and hydrogen damage forms.

According to the results of hydrogen permeation experiment, X80 pipeline steel showed the best hydrogen permeation performance under the condition of 20 mA/cm^2^ AC charging. Therefore, the effects of hydrogen charging in air and at 5 and 20 mA/cm^2^ on the mechanical properties of base metal and welded joint were systematically studied, and the variation law was analyzed by stress–strain curve. At the same time, the slow strain rate tensile tests of DC hydrogen charging and anode corrosion at the corresponding current density were carried out to compare the effects of cathode hydrogen charging and anode corrosion on the hydrogen damage behavior of the material during AC hydrogen charging.

## 3. Results and Discussion

### 3.1. Hydrogen Permeation Behavior of X80 Pipeline Steel

In this paper, the apparent hydrogen diffusion coefficient, hydrogen permeation flux J and underground hydrogen concentration C_0_ of X80 pipeline steel are calculated by the following formula:

(1) $$J=i_{\infty }/F$$

(2) $$D_{\mathrm{app}}=L^{2}/{6t}_{L}$$

(3) $$C_{0}=JL/D$$

where i∞ is the stable current density; F is the Faraday constant, 96,487.33 C/mol; L is the thickness of the sample; t_L_ is the lag time, which represents the time required for the anode current density to reach 63% of the stable current density.

**Figure 5:**
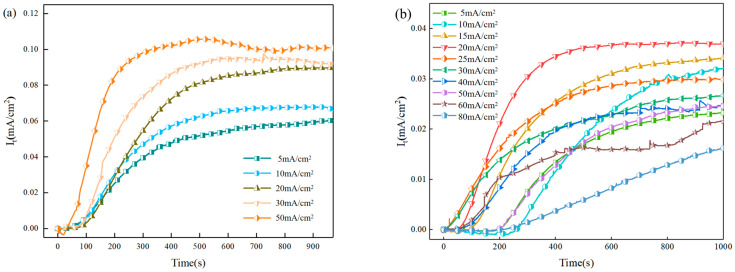
The hydrogen permeation curves of X80 pipeline steel under different hydrogen charging states are as follows: (**a**) DC hydrogen charging; (**b**) AC hydrogen charging.

Figure 5a shows the hydrogen permeation curve of X80 pipeline steel under direct current hydrogen charging. In order to reduce the influence of hydrogen defects in the sample on the experiment, we carried out two hydrogen permeation tests, and the results of the second hydrogen permeation were taken as the standard. Under the condition of direct current hydrogen charging, the hydrogen charging surface of the sample remained stable, and the hydrogen permeation curve showed a smooth curve. Figure 5b shows the hydrogen permeation curves under different AC-positive half-wave current hydrogen charging conditions, showing a similar trend to that under DC hydrogen charging, which is also shown as a smooth curve. The results show that when the AC is charged with hydrogen, the cathode part of the AC has little effect on the potential, resulting in that the curve change is mainly affected by the anode part of the AC. The anode part of the alternating current will cause corrosion on the surface of the sample and destroy the steady state of the surface, thus affecting the hydrogen permeation efficiency.

The specific values of the hydrogen permeation parameters are listed in Table 2. Under direct current (DC) charging, the hydrogen permeation flux of the X80 pipeline steel increases with the current density. This is because the applied cathodic current density serves as the primary external driving force for hydrogen entry. An increase in current density enhances this driving force, accelerating the hydrogen absorption rate at the input surface and establishing a steeper concentration gradient across the metal thickness. Consequently, the time required for the hydrogen permeation current to reach a steady state is shortened, resulting in a corresponding increase in the steady-state hydrogen permeation flux. When the AC is used for hydrogen charging, the hydrogen permeation flux increases with the increase in the current density, and reaches the maximum when the current density reaches 20 mA/cm^2^. At this time, the apparent hydrogen diffusion coefficient of X80 pipeline steel reaches the maximum. When the current density exceeds this critical value, the hydrogen permeation flux begins to decline. This phenomenon primarily stems from two contributing factors. Firstly, the enhanced anodic corrosion from the alternating current at high current densities leads to the formation of a substantial layer of dense corrosion products on the specimen surface, creating a physical barrier that impedes hydrogen atom penetration. Secondly, with the formation of these corrosion products, a significant pseudo-capacitance effect emerges. The continuous redox reaction of the Fe^2+^/Fe^3+^ couple and the repeated charging–discharging process of the electric double layer collectively divert a portion of the alternating current. This consequently reduces the effective current component actually available for the hydrogen evolution reaction. Therefore, the hydrogen permeation flux and apparent hydrogen diffusion coefficient of X80 pipeline steel decreased at a high current density of more than 20 mA/cm^2^, as shown in Figure 5b.

### 3.2. Hydrogen Diffusion Behavior of X80 Pipeline Steel

**Figure 6:**
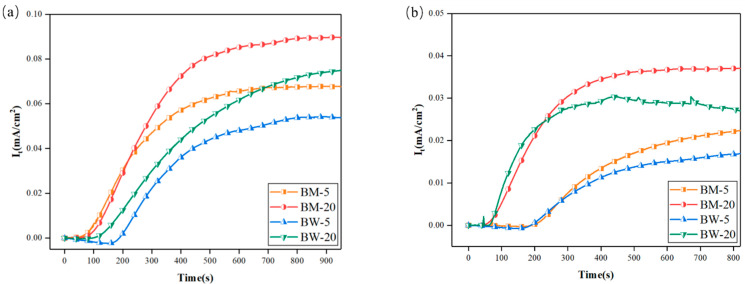
Hydrogen permeation curves of welded joints of X80 pipeline steel under different hydrogen charging states: (**a**) DC hydrogen charging; (**b**) AC hydrogen charging.

Figure 6 presents the hydrogen permeation curves of the X80 pipeline steel base metal and welded joint under different hydrogen charging conditions. A comparison of the hydrogen permeation behavior between the base metal and the welded joint was conducted at current densities of 5 and 20 mA/cm^2^. It was observed that the welded joint exhibited a consistent trend with the base metal: as the current density increased, the driving force for hydrogen atom permeation intensified. This led to an increased hydrogen permeation rate, a shorter time required to reach a steady state, and a corresponding increase in the hydrogen permeation flux.

However, compared to the base metal, the welded joint demonstrated a lower hydrogen permeation coefficient and a reduced steady-state hydrogen flux under the same current density. This discrepancy is primarily attributed to the high density of crystalline defects, such as dislocations and vacancies, within the welded joint. These defects act as efficient hydrogen traps, capturing and retaining diffusing hydrogen atoms, which significantly impedes their macroscopic transport. Consequently, the effective hydrogen diffusion flux is reduced.

### 3.3. Slow Strain Rate Tensile Test Results

Through electrochemical hydrogen charging and slow strain rate tensile tests, we studied the stress corrosion cracking behavior of X80 pipeline steel base metal and joints at 5 mA/cm^2^ and 20 mA/cm^2^. In this study, the elongation loss rate δ of the X80 pipeline steel sample was used to define the hydrogen embrittlement sensitivity index (EI). The calculation formula is as follows:

(4) $$\mathrm{EI}\;=\;\frac{\delta_{0}\;-\;\delta_{H}}{\delta_{0}}\;\times \;100\%$$

In the above analysis, δ_0_ represents the elongation of X80 pipeline steel without hydrogen charging, and δ_H_ represents the corresponding performance index after hydrogen charging. The increase in hydrogen embrittlement sensitivity index reflects the increase in hydrogen embrittlement sensitivity of materials.

**Figure 7:**
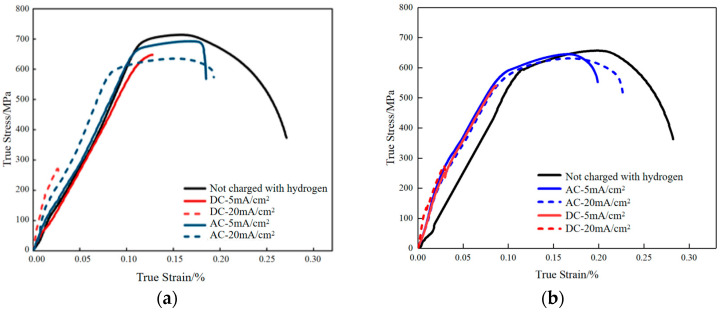
Stress–strain curves of X80 pipeline steel (**a**) base metal specimen; (**b**) welded joint under different hydrogen charging conditions.

Figure 7 shows the stress–strain response of X80 pipeline steel base metal and welded joint under different hydrogen charging conditions. The mechanical properties of welded joints are affected by many factors, including the heat input of the welding process, the chemical composition of the welding wire, the crystallization process, etc. [29]. Considering the data in Table 3 and Table 4, comprehensive analysis shows that the hydrogen charging environment affects the mechanical properties of both, but there are also some differences. In the base metal, both DC and AC hydrogen charging lead to the decrease in mechanical properties, and the increase in current density makes the decrease more significant. Under the condition of 20 mA/cm^2^, the elastic modulus of the base metal increases, but the tensile strength decreases. Specifically, the yield strength and tensile strength of DC hydrogen charging decreased by 62.04% and 11.01%, respectively, compared with the uncharged state, reflecting obvious hydrogen-induced damage. This is mainly due to the direct current to promote the rapid infiltration of hydrogen atoms, causing internal defects and microcracks. In contrast, although AC charging hydrogen has the strongest hydrogen permeation capacity at 20 mA/cm^2^, its anodic corrosion hinders the continuous infiltration of hydrogen, thereby reducing the risk of stress corrosion cracking. The welded joint shows a similar trend to the base metal after hydrogen charging, that is, the mechanical properties decrease with the increase in current density, but the performance decrease is generally smaller than that of the base metal under the same conditions. For example, under the condition of DC hydrogen charging, the yield strength and tensile strength of the welded joint decreased by 57.99% and 4.11% at 20 mA/cm^2^, respectively, which were lower than those of the base metal. In addition, at a low current density of 5 mA/cm^2^, the elastic deformation stage of the welded joint is almost unaffected, indicating that it is more resistant to low-density hydrogen charging environment. Under the condition of AC hydrogen charging, the welded joint also shows better mechanical properties, which further indicates that its corrosion resistance is better than that of the base metal.

From the perspective of hydrogen embrittlement sensitivity, both the base metal and the welded joint show the highest hydrogen embrittlement sensitivity under DC hydrogen charging conditions, while it is significantly reduced in the AC hydrogen charging environment. Although the overall corrosion resistance of the welded joint is better, the change trend of the two is consistent: DC hydrogen charging causes more serious hydrogen atom accumulation and microcrack formation, resulting in a significant decrease in mechanical properties and a severe loss of ductility. This marked reduction in elongation and reduction in area, as a prominent indicator of material embrittlement, aligns with the core phenomenon of ductility loss reported in aging studies of pipeline steels [33]; AC hydrogen charging effectively slows down hydrogen permeation due to the ‘self-inhibition’ effect of anode corrosion, thus reducing the sensitivity of hydrogen embrittlement. Therefore, the welded joint is not only superior to the base metal in terms of mechanical property retention, but also shows the same trend and better overall performance in terms of hydrogen embrittlement sensitivity control.

### 3.4. Fracture Analysis

**Figure 8:**
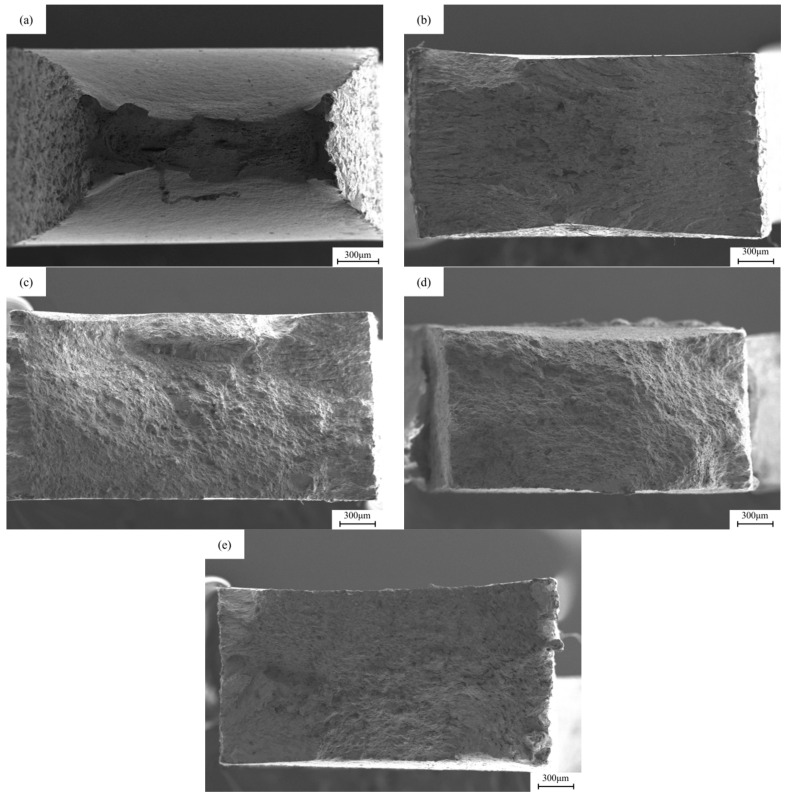
The macroscopic fracture morphology of X80 pipeline steel base metal samples under different hydrogen charging conditions: (**a**) uncharged; (**b**,**c**) under the DC of 5 mA/cm^2^ and 20 mA/cm^2^; (**d**,**e**) at 5 mA/cm^2^ and 20 mA/cm^2^ AC.

Figure 8 shows the SEM images of the macroscopic fracture morphology after slow strain stretching under different hydrogen charging conditions. In the sample without hydrogen charging, the cross-sectional area of the fracture is relatively small, while the cross-sectional necking is relatively large. After direct current hydrogen charging, the cross-sectional area shrinkage of the fracture after 5 mA/cm^2^ and 20 mA/cm^2^ current hydrogen charging is 9.25% and 1.6%, respectively, resulting in a significant decrease in the toughness of the material. After alternating current hydrogen charging, the material retains a certain toughness. The cross-sectional area shrinkage of the fracture after 5 mA/cm^2^ and 20 mA/cm^2^ current hydrogen charging is 31.89% and 15.16%, respectively, and the cross-sectional area shrinkage of the sample decreases. After a long period of hydrogen charging and corrosion treatment, the cross-sectional area of the fracture of the sample is larger than that of the uncharged state, showing that the fracture form of the material changes from toughness to brittleness.

**Figure 9:**
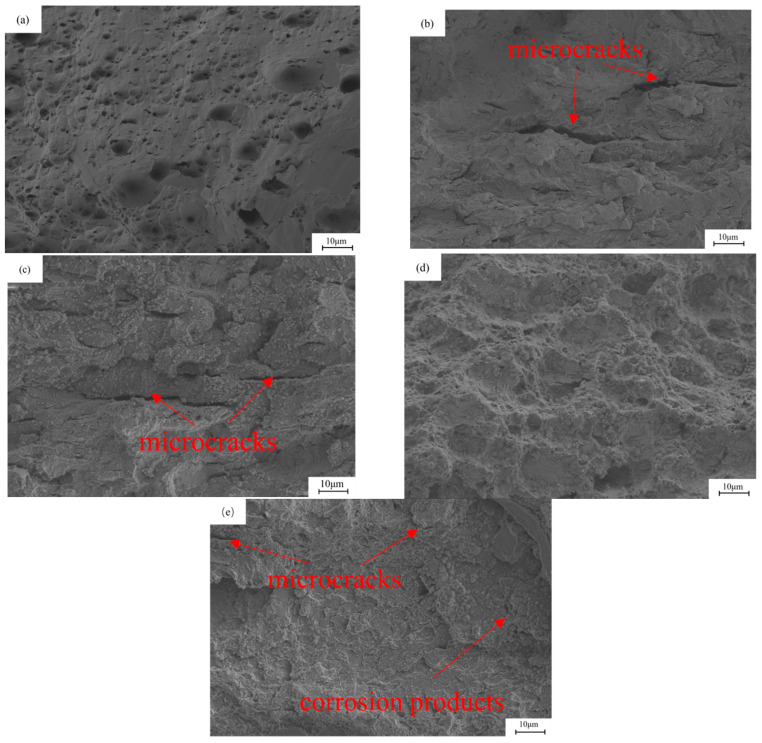
The fracture morphology of X80 pipeline steel base metal samples under different hydrogen charging conditions: (**a**) in air; (**b**,**c**) under the DC of 5 mA/cm^2^ and 20 mA/cm^2^; (**d**,**e**) at 5 mA/cm^2^ and 20 mA/cm^2^ AC.

**Figure 10:**
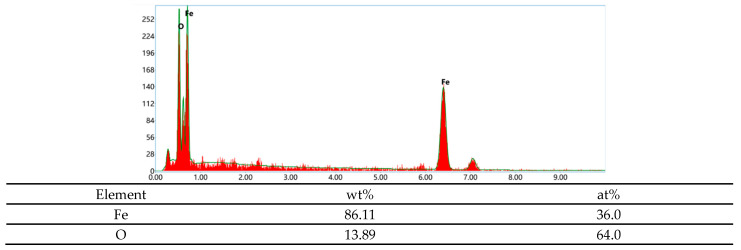
EDS elemental analysis of the corrosion products at the fracture of the base metal after hydrogen charging at 5 mA/cm^2^ AC.

Figure 9 shows the micro-graph of the fracture of the base metal under different hydrogen charging conditions. The samples before and after hydrogen charging show obvious fracture differences. Before hydrogen charging, the fracture shows typical dimple characteristics, containing a large number of small and dense dimples, showing typical ductile fracture. On the contrary, the fracture morphology of the sample after hydrogen charging changed significantly. After direct current hydrogen charging, the strength of the sample decreased significantly, and the fracture surface showed a flat shape with a weak necking. A large number of tiny cracks were observed. In addition, the fracture shows obvious brittle characteristics, especially in Figure 9b,c. Under the alternating current, the fracture surface of the sample is irregular, and the degree of necking is significantly greater. At the current density of 5 mA/cm^2^, the appearance of micro-pit characteristics was observed, which appeared together with the corrosion products. As shown in Figure 10, EDS analysis revealed that these corrosion products mainly consist of O and Fe elements with an atomic ratio of approximately 1.7:1, suggesting the predominant presence of Fe_2_O_3_ and FeOOH. When the current density is 20 mA/cm^2^, the fracture surface shows a flat morphology, showing a large number of corrosion products. The similar Fe/O atomic ratio (≈1.7:1) detected by EDS confirms that the corrosion layer is primarily composed of Fe_2_O_3_ and FeOOH. In addition, the fracture surface exhibits obvious brittle fracture characteristics, as shown in Figure 9d,e. In the AC hydrogen charging, the contact time between the sample and the solution is long, and the electrochemical corrosion is fully carried out, resulting in obvious anodic dissolution. The dissolved oxygen in the solution is depleted, and an oxygen-poor zone is formed at the contact between the sample surface and the solution, which inhibits the cathodic reaction. The bonding force between the metal lattices at the anodic dissolution is weakened, and the grain boundaries are damaged by electrochemical corrosion, and finally corrosion is formed. In this process, electrochemical corrosion plays a decisive role. As the stretching progresses, the electrochemical effect gradually weakens, and hydrogen damage and stress act together on the surface of the metal electrode. When the sample is corroded by electrochemical action, under the action of tensile stress, the grain boundary of the matrix inside the corrosion pit is destroyed, the lattice is distorted, and the microcracks begin to form from the corrosion pit, gradually increase and expand, and the sensitivity of stress corrosion cracking increases significantly.

**Figure 11:**
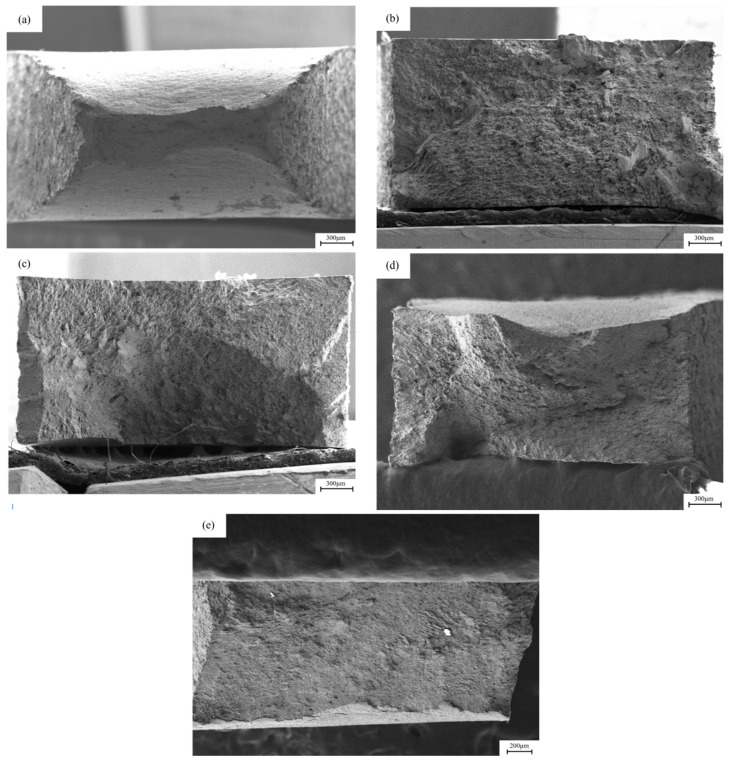
The macroscopic fracture morphology of X80 pipeline steel welded joint samples under different hydrogen charging conditions: (**a**) in air; (**b**,**c**) under the direct current of 5 mA/cm^2^ and 20 mA/cm^2^; (**d**,**e**) at 5 mA/cm^2^ and 20 mA/cm^2^ AC.

**Figure 12:**
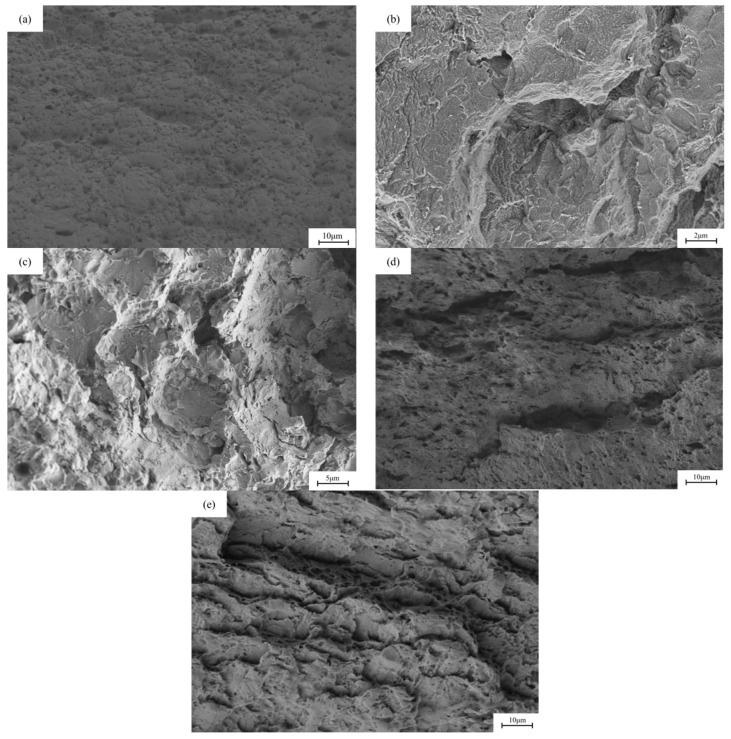
The fracture morphology of X80 pipeline steel welded joint samples under different hydrogen charging conditions: (**a**) in air; (**b**,**c**) under the direct current of 5 mA/cm^2^ and 20 mA/cm^2^; (**d**,**e**) at 5 mA/cm^2^ and 20 mA/cm^2^ AC.

Figure 11 and Figure 12 show the fracture morphology of X80 pipeline steel welded joints under different hydrogen charging conditions. The uneven structure caused by the welding process leads to a large number of defects such as inclusions, dislocations and vacancies in the lattice of the welded joint, which reduces the intergranular bonding force and increases the lattice activity. Under the stress of sufficient strength, lattice distortion may occur on the grain boundary surface of the welded joint, and this effect takes the shortest time, so the stress corrosion cracking sensitivity of the welded joint is higher. It can be observed that the degree of cross-section necking of the sample is significantly reduced after direct current hydrogen charging. After DC hydrogen charging at current densities of 5 mA/cm^2^ and 20 mA/cm^2^, the cross-sectional area shrinkage of the fracture was 0.54% and 0.15%, respectively, showing a large toughness reduction. After hydrogen charging by alternating current, the material still maintains a certain toughness. After hydrogen charging at current densities of 5 mA/cm^2^ and 20 mA/cm^2^, the cross-sectional area shrinkage of the fracture is 28.85% and 20.11%, respectively, and the cross-sectional area shrinkage of the sample decreases.

In the process of AC hydrogen charging, cathodic polarization is applied to the applied potential, so that the cathodic reaction rate is higher than the anodic reaction rate, which effectively inhibits local metal dissolution and plays an inhibitory role in metal corrosion. However, since the hydrogen produced by the cathode reaction enters the steel during the diffusion process and accumulates at the defect site (hydrogen trap), when the hydrogen concentration reaches a certain critical value, it will lead to hydrogen-induced cracking, which in turn induces crack propagation inside the material. With the increase in cathode current density, the cathodic hydrogen charging reaction is continuously strengthened, resulting in an increase in hydrogen entering the steel, which increases the tendency of hydrogen-induced cracking, thereby causing stress corrosion cracking and increasing its sensitivity. In the case of direct current hydrogen charging, the metal surface undergoes cathodic polarization reaction, and the corrosion is inhibited, but the cathodic reaction continues, resulting in a continuous flow of hydrogen atoms into the metal to form hydrogen damage. In this case, the accumulation of hydrogen causes stress corrosion cracking, which makes the fracture show obvious brittle morphology, characterized by terrace-like features and river patterns, while the air-tested specimen exhibits micro-voids and peaks, confirming a transition from ductile to brittle fracture [34].

## 4. Conclusions

(1) Under DC hydrogen charging, the hydrogen permeation flux of X80 pipeline steel increases with current density, indicating stronger hydrogen permeation. Under AC charging, the flux peaks at 20 mA/cm^2^ then decreases. High-frequency polarization promotes permeation, but anode corrosion and surface products hinder hydrogen entry. Welded joints show better mechanical properties under AC charging but remain sensitive to DC-induced stress corrosion cracking.
(2) SSRT results show hydrogenation and corrosion degrade X80 pipeline steel, shifting from ductile to brittle fracture. This fracture mode transition is directly validated by fractographic analysis, which reveals a change from dimpled structures to quasi-cleavage features. DC hydrogen charging increases stress corrosion cracking sensitivity in both base metal and welded joints. AC charging improves hydrogen permeability, while corrosion morphology reduces crack propagation and sensitivity.

## Figures and Tables

**Table 1:** Chemical composition of X80 pipeline steel (wt%).

Element	C	Mn	Si	P	S	Ni	Cu
wt%	0.463	1.94	0.28	0.07	0.008	0.08	0.002
element	Mo	Al	Ti	Nb	N	Cr	Fe
wt%	0.2	0.07	0.04	0.07	0.013	0.29	Balance

**Table 2:** Hydrogen permeation parameters of X80 pipeline steel under different hydrogen charging states.

i_c_ (mA/cm^2^)	J (10^−10^ mol/(s·cm^2^))		D (10^−6^ cm^2^/s)		C_0_ (10^−6^ cm^−3^)	
	DC	AC	DC	AC	DC	AC
5	6.40	2.68	5.68	3.53	11.26	7.59
10	7.02	4.01	6.25	2.73	11.24	14.66
15		3.69		4.97		7.43
20	9.29	3.85	5.40	7.62	17.22	5.05
25		3.12		6.81		4.58
30	10.10	2.77	7.03	6.12	14.37	4.52
40		2.66		5.19		5.13
50	10.96	2.64	11.22	3.62	9.77	7.28
60		2.59		1.88		13.75
80		2.68		3.53		7.59

**Table 3:** Mechanical property parameters of X80 pipeline steel base metal under different hydrogen charging conditions.

Stretch Environment	Air	DC/(mA/cm^2^)		AC/(mA/cm^2^)	
		5	20	5	20
Tensile Strength, σb (MPa)	713 ± 10	647 ± 10	270 ± 5	692 ± 10	635 ± 10
Elongation, δ (%)	8 ± 1	4 ± 1	1 ± 0.2	6 ± 1	5 ± 1
Reduction in Area, ψ (%)	75 ± 10	9 ± 3	2 ± 1	31 ± 5	15 ± 3
EI/%	/	52 ± 5	90 ± 5	30 ± 5	33 ± 5

**Table 4:** Mechanical properties results of X80 pipeline steel welded joint samples under different hydrogen charging conditions.

Stretch Environment	Air	DC/(mA/cm^2^)		AC/(mA/cm^2^)	
		5	20	5	20
Tensile Strength, σb (MPa)	657 ± 10	540 ± 10	276 ± 10	645 ± 10	630 ± 10
Elongation, δ (%)	9 ± 1	3 ± 0.5	1 ± 0.5	6 ± 1	7 ± 1
Reduction in Area, ψ (%)	83 ± 5	0.5 ± 0.2	0.2 ± 0.1	28 ± 2	20 ± 2
EI/%	/	69 ± 5	89 ± 5	29 ± 3	19 ± 3

## Data Availability

The original contributions presented in this study are included in the article. Further inquiries can be directed to the corresponding authors.
